# A prospective biomarker analysis of alvocidib followed by cytarabine and mitoxantrone in MCL-1-dependent relapsed/refractory acute myeloid leukemia

**DOI:** 10.1038/s41408-021-00568-3

**Published:** 2021-10-30

**Authors:** Joshua F. Zeidner, Tara L. Lin, Carlos E. Vigil, Gil Fine, M. Yair Levy, Aziz Nazha, Jordi Esteve, Daniel J. Lee, Karen Yee, Andrew Dalovisio, Eunice S. Wang, Juan M. Bergua Burgues, Jeffrey Schriber, Mark R. Litzow, Olga Frankfurt, Teresa Bernal Del Castillo, Vijaya Raj Bhatt, Bhavana Bhatnagar, Priyanka Mehta, Richard Dillon, Maria Vidriales Vicente, Stephen Anthony, David Bearss, Pau Montesinos, B. Douglas Smith

**Affiliations:** 1 University of North Carolina, Lineberger Comprehensive Cancer Center, Chapel Hill, NC USA; 2 University of Kansas Medical Center, Kansas City, KS USA; 3 University of Iowa, Iowa City, IA USA; 4 Robert H. Lurie Comprehensive Cancer Center, Northwestern University Feinberg School of Medicine, Chicago, IL USA; 5 Sumitomo Dainippon Pharma Oncology, Lehi, UT USA; 6 Texas Oncology-Baylor Charles A. Sammons Cancer Center, Dallas, TX USA; 7 Cleveland Clinic, Taussig Cancer Institute, Cleveland, OH USA; 8 Hospital Clinic de Barcelona, Universitat de Barcelona, Barcelona, Spain; 9 Columbia University Irving Medical Center, New York, NY USA; 10 Princess Margaret Cancer Centre, Toronto, ON Canada; 11 Ochsner Medical Center, New Orleans, LA USA; 12 Roswell Park Comprehensive Cancer Center, Buffalo, NY USA; 13 Hospital San Pedro de Alacantra, Caceres, Spain; 14 Cancer Treatment Centers of America, Phoenix, AZ USA; 15 Mayo Clinic, Rochester, MN USA; 16 Hospital Universitario Central de Asturias, Asturias, Spain; 17 Fred and Pamela Buffet Cancer Center, University of Nebraska Medical Center, Omaha, NE USA; 18 The Ohio State University Comprehensive Cancer Center, Columbus, OH USA; 19 University Hospitals Bristol, Bristol, UK; 20 Department of Medical and Molecular Genetics, King’s College, London, UK; 21 Hospital Clinico Universitario de Salamanca, Salamanca, Spain; 22 University of Utah, Salt Lake City, UT USA; 23 Hospital Universitario Politecnico La Fe, Valencia, Spain; 24 Sidney Kimmel Comprehensive Cancer Center, Johns Hopkins School of Medicine, Baltimore, MD USA

**Keywords:** Phase II trials, Drug development, Drug development, Acute myeloid leukaemia

**Dear Editor**,

Overall outcomes are dismal in patients with relapsed/refractory (R/R) acute myeloid leukemia (AML). Alvocidib is a multi-cyclin-dependent kinase (multi-CDK) inhibitor with potent activity against CDK9. CDK9 forms a complex with cyclin T1, positive transcription elongation factor b, which exists in a superenhancer complex to regulate the activity of RNA-polymerase II. By inhibiting CDK9, alvocidib leads to the suppression of RNA-polymerase II-mediated transcription of myeloid cell leukemia-1 (MCL-1), a pro-survival BCL-2 family member that inhibits the intrinsic pathway of apoptosis and promotes leukemia survival [1]. MCL-1 has a short half-life and is dependent on continuous transcription from RNA-polymerase II for activity [2]. There is a strong rationale to investigate targeted strategies of MCL-1 inhibition in diverse AML treatment settings.

Alvocidib followed by cytarabine and mitoxantrone (ACM) has been investigated in a timed-sequential therapy approach with the purpose of priming leukemia cells to undergo apoptosis during opportune time periods of leukemia cell-cycle progression with cell-cycle-specific anti-leukemia agents. Serial studies in both newly diagnosed (*n* = 256) and R/R AML (*n* = 149) revealed encouraging findings with ACM though notably some of these studies included patients aged >65 years and did not prospectively assess for MCL-1 dependence [3]. We hypothesized that leukemia dependence of MCL-1 may predict for response to ACM. We conducted a two-stage clinical trial of ACM in MCL-1-dependent R/R AML. Stage 1 was a biomarker-based prospective analysis of ACM activity based on MCL-1 dependence in R/R AML and newly diagnosed AML. Stage 2 was a randomized phase 2 trial of ACM vs. cytarabine and mitoxantrone in MCL-1-dependent R/R AML to assess whether alvocidib improves composite complete remission (CRc) rates in R/R AML. However, slow accrual and drug availability led to the early termination of the study. Herein we focus on the results of the completed MCL-1-dependent biomarker analysis of ACM in R/R AML.

Zella-201 (ClinicalTrials.gov identifier: NCT02520011) enrolled adults 18–65 years with pathologically confirmed AML in first relapse (CR duration <24 months) or with refractory AML after 1–2 cycles of intensive induction therapy. MCL-1 dependence was assessed using the pro-apoptotic, BH3-sensitizing, NOXA-mimetic peptide, T-MS1, as previously described [4], and initially defined as ≥40% (further details in Supplemental Appendix). In addition, an exploratory cohort of newly diagnosed high-risk (NDHR) AML enrolled patients with MCL-1 dependence (≥40%) (see Supplemental Appendix). Treatment plan is outlined in Fig. 1A–B. The primary endpoint of this study was the proportion of R/R AML patients with MCL-1 dependence ≥40% achieving CRc (CR/CR with incomplete recovery (CRi)) after cycle 1 of therapy with ACM (see Supplemental Appendix). Full eligibility criteria are outlined in Supplemental Appendix. This study was conducted in accordance with the Declaration of Helsinki after approval by ethics committee of each participating center.

**Fig. 1:**
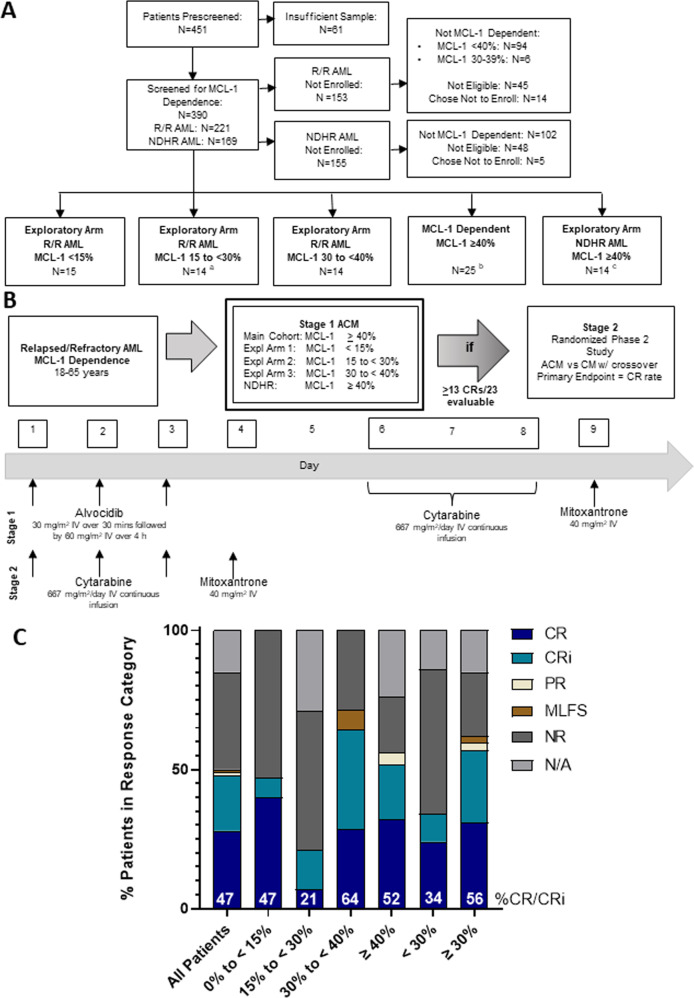
Study diagram, consort diagram, and response rate. **A** Consort diagram. Not evaluable for response: ^a^Did not receive mitoxantrone due to death (enterocolitis and sepsis, *n* = 1); ^b^Suicide attempt prior to response assessment (*n* = 1) and acute decompensation due to septic shock prior to completion of cytarabine (*n* = 1); ^c^Did not complete alvocidib cycle 1, day 2 due to TLS or day 3 due to tachycardia, tachypnea, hypotension, and worsening liver function tests. Patient eventually withdrew consent (*n* = 1). **B** Study diagram. **C** Response rates of alvocidib in combination with cytarabine and mitoxantrone in relapsed/refractory AML. Percentages within each column indicate response in that cohort. CRi CR with incomplete recovery, PR partial remission, MLFS morphologic leukemia-free state, NR no response, N/A no response assessment due to early death.

Between January 2016 and December 2019, 451 patients (R/R AML: *n* = 221; NDHR AML: *n* = 169) were screened for MCL-1 dependence (consort diagram, Fig. 1A). The overall proportion of AML patients initially determined to be MCL-1 dependent (≥40%) was 39% (R/R AML: 84/221 = 38%, NDHR AML: 67/169 = 40%). The threshold to define MCL-1 dependence was later amended to ≥30% and the overall proportion of R/R AML patients with MCL-1 dependence ≥30% was 47% (104/221). Eighty-two patients were enrolled onto one of the five cohorts: (1) MCL-1 <15% (*n* = 15), (2) MCL-1: 15–<30% (*n* = 14), (3) MCL-1: 30–<40% (*n* = 14), (4) MCL-1: ≥40% (*n* = 25), and (5) NDHR AML with MCL-1 ≥40% (*n* = 14). Patient characteristics are shown in Table 1. Among all R/R AML patients (*n* = 68), the CR, CRi, and CRc rates were 28, 19, and 47%, respectively (Fig. 1C). Three patients were not evaluable for response (Fig. 1A). A comparison of all patients (intent-to-treat) and response-evaluable patients in each cohort is shown in Supplemental Tables 1 and 2. Among all patients, the CRc rate was 47% (7/15), 21% (3/14), 64% (9/14), and 52% (13/25) in R/R AML patients with MCL-1 <15%, 15–<30%, 30–<40%, and ≥40%, respectively. Given the similar clinical activity of ACM in MCL-1 ≥30% and ≥40%, the definition of MCL-1 dependence was subsequently amended to MCL-1 ≥30%. CRc rate was 56% in patients with R/R AML with MCL-1 ≥30% vs. 34% in R/R AML with MCL-1 <30% (*P* = 0.08). Notably, refractory AML patients with MCL-1 dependence had a CRc rate of 52% compared with 38% in refractory AML patients without MCL-1 dependence (Supplemental Fig. 1). In the NDHR AML cohort (*n* = 14), the CR, CRi, and CRc rates among all patients were 43, 14, and 57%, respectively.

**Table 1:** Stage 1 patient characteristics.

Patient characteristics	Exploratory cohort 1: MCL-1: <15% (*N* = 15)	Exploratory cohort 2: MCL-1: 15–<30% (*N* = 14)	Exploratory cohort 3: MCL-1: 30–<40% (*N* = 14)	Main cohort: MCL-1: ≥40% (*N* = 25)	Overall relapsed/refractory (*N* = 68)	Exploratory cohort 4: NDHR: MCL-1: ≥40% (*N* = 14)
Age, median (range), years	46 (26, 61)	50 (39, 64)	55 (24, 62)	52 (26, 65)	56 (24, 65)	53 (22, 65)
Age ≥60, years (%)	3 (20)	5 (36)	6 (43)	6 (24)	20 (29)	6 (43)
Male, *n* (%)	5 (33)	11 (79)	9 (64)	9 (36)	34 (50)	11 (79)
Refractory/ER/LR, *n* (%)	8 (53)/4 (27)/3 (20)	5 (36)/6 (43)/3 (21)	9 (64)/3 (21)/2 (14)	12 (48)/8 (32)/5 (20)	34 (50)/21 (31)/13 (19)	0 (0)/0 (0)/0 (0)
Prior stem cell transplant	3 (20)	4 (29)	3 (21)	4 (16)	14 (21)	2 (14)
ECOG performance 0/1/2, *n* (%)	7 (47)/8 (53)/0 (0)	5 (36)/6 (43)/3 (21)	7 (50)/7 (50)/0 (0)	13 (52)/9 (36)/2 (8)	32 (47)/30 (44)/6 (9)	5 (36)/7 (50)/2 (14)
Bone marrow blasts (%), median (range)	51 (18, 88)	35 (5, 77)	30 (8, 92)	47 (7, 92)	47 (5, 92)	37 (9, 83)
Baseline WBC (×10^9^/L), median (range)	4 (1, 33)	2 (0.2, 55)	2 (1, 26.6)	5 (2, 46)	2 (0.2, 55)	2 (0.4, 10)
Secondary AML, *n* (%)	1 (7)	2 (14)	1 (7)	3 (12)	7 (10)	10 (71)
t-AML/PMM, *n* (%)	0 (0)/1 (7)	0 (0)/2 (14)	0 (0)/1 (7)	0 (0)/3 (12))	0 (0)/7 (10)	1 (7)/9 (64)
AML with MRC, *n* (%)	6 (40)	5 (36)	5 (36)	6 (24)	22 (32)	11 (79)
Genomically defined secondary AML, *n* (%)	3 (20.0)	1 (7)	4 (29)	5 (20)	13 (19)	4 (29)
ELN classification, *n* (%)						
Favorable/intermed/adverse	2 (13)/4 (27)/9 (60)	2 (14)/3 (21)/6 (43)	0 (0)/8 (57)/6 (43)	1 (4)/9 (36)/11 (44)	5 (7)/24 (35)/32 (47)	3 (21)/2 (14)/9 (64)
SWOG cytogenetics, *n* (%)						
Favorable	0 (0)	0 (0)	0 (0)	1 (4)	1 (1)	1 (7)
Intermediate	7 (47)	3 (21)	4 (29)	11 (44)	25 (37)	4 (29)
Unfavorable	7 (47)	6 (43)	6 (43)	6 (24)	25 (37)	9 (64)
Unknown	1 (7)	2 (14)	4 (29)	3 (12)	10 (15)	0 (0)

*CR* complete remission, *CRi* complete remission with incomplete recovery, *ECOG* Eastern Cooperative Oncology Group, *ELN* European LeukemiaNet, *ER* early relapse, *Intermed* intermediate, *LR* late relapse, *MCL-1* myeloid cell luekemia-1, *MRC* myelodysplasia-related changes, *NDHR* newly diagnosed high risk, *PMM* prior myeloid malignancy, *PR* partial remission, *SWOG* Southwestern Oncology Group, *t-AML* treatment-related AML, *WBC* white blood cells.

For all R/R AML cohorts, median follow-up, overall survival (OS), event-free survival (EFS), and relapse-free survival (RFS) was 7.2 months, 10.0 months [95% confidence interval (CI): 5.9, 16.9 months], 2.5 months [95% CI: 1.6, 3.9 months] and 11.8 months [95% CI: 6.0, 17.5 months], respectively (Supplemental Fig. 2). One- and 2-year OS was 48 and 24%, respectively. A comparison of clinical outcomes among patients with MCL-1-dependent R/R AML and non-MCL-1-dependent R/R AML patients is outlined in Supplemental Tables 3–5. Median OS was 11.2 vs. 7.4 months in those with R/R MCL-1 dependence vs. R/R AML without MCL-1 dependence, respectively (*P* = 0.40).

Treatment-emergent grade ≥3 non-hematologic adverse events after alvocidib are illustrated in Supplemental Table 5. Overall 30-day mortality was 6% in R/R AML and 0% in NDHR AML. In those who achieved CRc, median time to full neutrophil (absolute neutrophil count ≥1.0 × 10^9^/L) recovery for R/R was 48 [48, 71] days compared with 45 [40, NA] days in the NDHR AML cohort.

A gene matrix of baseline mutations and genomic classification stratified based on response and treatment setting to ACM is shown in Supplemental Fig. 3 (R/R: *n* = 67; NDHR: 13). In order to assess whether MCL-1 dependence may enrich for overall response to ACM, bootstrap resampling analysis was performed in order to infer population variance of MCL-1 priming results (Supplemental Appendix) [5]. There was a significantly higher frequency of overall response observed in the MCL-1-dependent cohort (MCL-1 ≥30%) compared with non-responders (*P* < 0.001), whereas overall response was less frequently observed in the MCL-1 <30% cohort compared with non-responders (Supplemental Fig. 4).

This is the first clinical trial prospectively assessing MCL-1 dependence as a biomarker for AML. We have shown that 47% of R/R AML patients are MCL-1 dependent (≥30%) and may be candidates for MCL-1-directed therapies [3]. This study met the primary endpoint with a CRc rate of 57% in MCL-1-dependent (≥40%) R/R AML. In order to broaden the putative patient population who may benefit from ACM, the criteria for MCL-1 dependence was amended to ≥30%. Overall CRc rates were non-significantly higher in patients with MCL-1 ≥30% vs. MCL-1 <30% (56 vs. 34%; *P* = 0.08). However, the study design was not powered to compare CRc rates between MCL-1-dependent cohorts and small numbers of patients likely precluded statistical significance. Salutary clinical activity was also evident in a high-risk MCL-1-dependent newly diagnosed AML exploratory cohort (CRc = 62%), which appear to be similar to ACM in unselected newly diagnosed poor-risk AML patients [3, 6].

Despite recent treatment advances in R/R AML, there remains no standard-of-care salvage regimen, and R/R AML continues to be the highest unmet need in AML. We divided R/R AML patients into 3 distinct subgroups: refractory (induction failure or CR1 duration <3 months), early relapse (CR1 duration 3–12 months), and late relapse (CR1 duration 12–24 months). The majority of R/R AML patients had either refractory or early relapse (84%) compared with late relapse (16%) corroborating the poor-risk group of patients enrolling on this study. In fact, median OS was 16.9 months in patients with refractory MCL-1-dependent AML, which compares favorably to outcomes seen with conventional chemotherapy agents in refractory AML.

Previous studies have demonstrated clinical activity of ACM in R/R AML. A randomized phase 2 study performed by the Eastern Cooperative Oncology Group (ECOG) investigated three treatment regimens for R/R AML: ACM, sirolimus plus mitoxantrone, etoposide, cytarabine, and carboplatin plus topotecan. Of the three regimens studied, ACM was the only regimen to meet the primary endpoint of the study with a CR rate of 28% [7]. A composite of 113 R/R AML patients treated with ACM across 3 single-arm phase 1 and 2 trials revealed an overall CRc rate of 38% with unsurprisingly lower responses in refractory compared with relapsed AML (CRc rates 14 vs. 72%, respectively) [3]. Importantly, these studies did not differentiate MCL-1 dependence among those treated with ACM. In MCL-1-dependent patients with R/R AML on the current study, overall CRc rates were 52 and 55% in refractory and early relapse AML, respectively, substantiating clinical activity in these poor-risk patient subgroups and comparing favorably to historical controls.

MCL-1 is an anti-apoptotic member of the BCL-2 family that inhibits BAX/BAK-mediated mitochondrial permeabilization and cell death. MCL-1 is upregulated in AML patients, particularly during relapse, and is a major contributor to AML progression [8, 9]. Leukemia cells utilize variable levels of BCL-2 anti-apoptotic proteins to undergo cellular proliferation and mitigate cell death through the intrinsic pathway of apoptosis. BH3 profiling assesses the relative cellular dependence of BCL-2 and/or MCL-1 for survival [10, 11] and represents a promising strategy to discriminate therapeutic response to BCL-2 mimetics [12]. While it is not possible to assess whether higher MCL-1 scores was associated with response to ACM in the context of this study, our findings suggest that alvocidib may have preferential clinical activity in MCL-1-dependent AML. Venetoclax has been investigated in combination with cytotoxic chemotherapy agents with encouraging clinical activity in newly diagnosed and R/R AML [13, 14] suggesting that combining agents targeting apoptotic pathways with cytotoxic chemotherapy regimens may be an effective therapeutic strategy. Our findings provide the foundation for further biomarker-driven strategies for BCL-2 mimetics in R/R AML.

In conclusion, ACM showed an acceptable safety profile and demonstrated clinical activity in MCL-1-dependent R/R AML. This is the first prospective trial incorporating a novel BH3 profiling biomarker-based strategy to identify whether MCL-1 dependence can predict for response to CDK9 inhibition. Our findings suggest that prospective analysis of MCL-1 dependence is feasible and can potentially stratify patients into unique biologic subgroups, which may be applicable to other tumor subsets with high dependence on MCL-1 for survival. Future biomarker-based study designs are warranted to determine predictive signatures of response to therapeutic agents targeting BCL-2 family members and other regulators of apoptosis.

## Supplementary information

Supplemental Methods
