# Supplementary material for: A prospective biomarker analysis of alvocidib followed by cytarabine and mitoxantrone in MCL-1-dependent relapsed/refractory acute myeloid leukemia
Source: Blood Cancer J. 2021 Oct 30;11(10):175. doi: 10.1038/s41408-021-00568-3 (PMC8557202; doi:10.1038/s41408-021-00568-3)
Supplement: Supplementary file 1 — Supplemental Methods [file 41408_2021_568_MOESM1_ESM.docx]

**Inclusion Criteria**

To be eligible for participation in the study, patients must meet all of the following inclusion criteria:

*During Prescreening:*

1. Be between the ages of ≥18 and ≤65 years
2. Have an established, pathologically confirmed diagnoses of acute myeloid leukemia (AML) by World Health Organization (WHO) criteria excluding acute promyelocytic leukemia (APL-M3) with a bone marrow of >5% blasts based on histology or flow cytometry
3. Be in first relapse (within 24 months of complete remission (CR)) or have failed induction therapy* (no CR or CR with incomplete recovery (CRi) after treatment with an intensive regimen [eg, anthracycline/cytarabine ± etoposide, gemtuzumab ozogamicin, or cladribine])

*Induction therapy may involve 1 or 2 cycles of the same regimen. Efficacy assessment of induction therapy must be >21 days from the start of the previous induction cycle.

1. Demonstrate MCL-1 dependence of ≥30% by mitochondrial profiling in bone marrow

*During Screening:*

1. Have an Eastern Cooperative Oncology Group (ECOG) performance status (PS) ≤2
2. Have a serum creatinine level ≤1.8 mg/dL
3. Have an alanine aminotransferase (ALT)/aspartate aminotransferase (AST) level ≤5 times upper limit of normal (ULN)
4. Have a total bilirubin level ≤2.0 mg/dL (unless secondary to Gilbert syndrome, hemolysis, or leukemia)
5. Have a left ventricular ejection fraction (LVEF) >45% by echocardiogram (ECHO) or multigated acquisition (MUGA) scan
6. Be nonfertile or agree to use an adequate method of contraception. Sexually active patients and their partners must use an effective method of contraception associated with a low failure rate during and for at least 6 months after completion of study therapy.
7. Be able to comply with the requirements of the entire study
8. Provide written informed consent prior to any study related procedure (In the event that the patient is re-screened for study participation or a protocol amendment alters the care of an ongoing patient, a new informed consent form must be signed.)

**Exclusion Criteria**

Patients meeting any one of these exclusion criteria will be prohibited from participating in this study.

1. Received more than 2 cycles of induction therapy for AML. Investigational agents as part of front-line therapy for AML may by acceptable following discussion with the Medical Monitor. Hydroxyurea is permitted (see #5 below).
2. Received any previous treatment with alvocidib or any other cyclin dependent kinase (CDK) inhibitor
3. Received a hematopoietic stem cell transplant within the previous 2 months
4. Have clinically significant graft versus host disease (GVHD), or GVHD requiring initiation or escalation of treatment within the last 21 days
5. Require concomitant chemotherapy, radiation therapy, or immunotherapy. Hydroxyurea is allowed up to the evening before starting (but not within 12 hours) of starting treatment on either arm.
6. Received >360 mg/m^2^ equivalents of daunorubicin
7. Have a peripheral blast count of >30,000/mm^3^ (may use hydroxyurea as in #5 above)
8. Received antileukemic therapy within the last 3 weeks (with the exception of hydroxyurea or if the patient has definite refractory disease).  Refractory patients who received therapy within the last 3 weeks may be eligible with prior approval of the Medical Monitor.
9. Diagnosed with acute promyelocytic leukemia (APL, M3)
10. Have active central nervous system (CNS) leukemia
11. Have evidence of uncontrolled disseminated intravascular coagulation
12. Have an active, uncontrolled infection
13. Have other life-threatening illness
14. Have other active malignancies or diagnosed with other malignancies within the last 6 months, except nonmelanoma skin cancer or cervical intraepithelial neoplasia
15. Have mental deficits and/or psychiatric history that may compromise the ability to give written informed consent or to comply with the study protocol.
16. Are pregnant and/or nursing
17. Have received any live vaccine within 14 days prior to first study drug administration

An exploratory cohort of newly diagnosed high-risk AML was included and defined by one of the following:

1) treatment-related AML after previous cytotoxic/radiation therapy

2) secondary AML from preexisting myelodysplastic syndrome or myeloproliferative neoplasm

3) adverse-risk cytogenetics as defined by European Leukemia Net Guidelines.^1^

**Methods:**

*MCL-1 Assessment:*MCL-1 dependence was assessed using the pro-apoptototic, BH3-sensitizing, NOXA-mimetic peptide, T-MS1, as previously described.^2^ MS1 primes cells to undergo apoptosis that are dependent on MCL-1 for survival. T-MS1 uses modifications to MS1 that allow for improved cell penetrations; T-MS1 has higher affinity and specificity than the MCL-1 targeting reagent Noxa BH3.^3^ Bootstrap resampling with replacement (1,000 replications) was performed to assess MCL-1 priming variation in responders versus non-responders.^4^ Two patient populations were assessed based on MCL-1 screening numbers: MCL-1 ≥30% (n=43) and MCL-1 <30% (n=25).

*Treatment Plan:*

Prophylactic antibacterial (levofloxacin or equivalent) and antiviral (valacyclovir or equivalent) agents were administered to all patients. Anti-fungal prophylactics were administered per institutional and investigator discretion. Mitoxantrone was omitted in subsequent cycles (after induction) if lifetime daunorubicin exposure >460 mg/m^2^, left ventricular ejection fraction <45%, or per investigator discretion. Those with refractory disease after cycle 1 of treatment were removed from the study. A second cycle of ACM was permitted in patients who achieved a overall response (CR, CRi, or partial remission) and in those who achieved clinical benefit with 1^st^ cycle without grade 4 toxicities. Allogeneic stem cell transplant (alloSCT) was permitted after treatment per investigator discretion.

*Tumor Lysis Syndrome:*

Patients received supportive care measures to prevent TLS with allopurinol 300 mg daily, oral phosphate binders, and 100 cc/hour of IV fluids at least 10 hours prior to treatment initiation until at least day 4 of therapy. Patients deemed to be at high-risk for tumor lysis syndrome (TLS; i.e. presence of extramedullary AML, white blood cell count >10x10^9^/L, bone marrow cellularity >50%, monocytic immunophenotype or those with *FLT3* mutations) received TLS labs every 2 hours for the first 24 hours. All other patients received TLS labs every 4 hours for the first 24 hours of treatment. Supportive care measures were recommended for patients with increasing potassium levels >4.0 mEq/L during alvocidib treatment.

*Disease Assessment:*

Bone Marrow (BM) aspirate and biopsy were performed at the time of full hematologic recovery (absolute neutrophil count >1x10^9^/L and platelet count >100x10^9^/L) or by day 45 of therapy. Response to therapy was assessed by 2010 European Leukemia Net (ELN) Guidelines.^5^ Those who achieved a partial remission (PR), CR or CR with incomplete recovery (CRi) were eligible to receive up to 3 cycles of consolidation with ACM at the same dose and schedule.

*Statistical Design:*

The primary endpoint of this study was the proportion of R/R AML patients with MCL-1 dependence >40% achieving composite CR (CRc: CR/CRi) after cycle 1 of therapy with ACM. Patients were stratified into three different subgroups based on disease status: 1) Refractory: persistent AML after 1-2 cycles of induction therapy or CR1 duration <90 days, 2) Early Relapse: first relapse with CR1 duration 90 days-1 year, 3) Late Relapse: first relapse with CR1 duration 1-2 years. The target sample size of 23 patients for Stage 1 was selected because it allows estimation of the CRc rate by a 90% confidence interval with maximum width of ±17%. Although this study does not follow a Simon 2-stage design, the study design had 80% power for testing the null hypothesis that the CRc rate is 50% against a one-sided alternative at the 5% level of significance when the actual CRc rate is 70%. If ≥13/23 patients enrolled in Stage 1 achieved CR/CRi, then this study would proceed to Stage 2.

**Mutational Analysis**

The TPI-ALV-201 protocol required that all patients have a bone marrow sample collected within 2 weeks of the first dose of Alvocidib. The sample was analyzed for AML diagnosis and cytogenetic profiling. For the TPI-ALV-201 study a central laboratory with a specific profiling panel was not utilized. Cytogenetic profiling was performed and reported according to each participating institution’s standard.

In order to be able to analyze the data from the composite of mutational panels from multiple institutions, a list of the most common tests of interest for the protocol were provided in the clinical database. The site then indicated if the listed mutation was present or absent for that patient based on the report from the institution’s standard laboratory. In addition, redacted pathology and cytogenetic reports were uploaded into the database for review by the Sponsor’s medical monitor. The medical monitor completed a 100% review of the data against the reports to assure the recording of the mutations was accurate.

**Supplementary Figure 1.** Response of early relapse (ER: CR duration 3-12 months), late relapse (LR: CR duration 12-24 months), and refractory (Ref: refractory to 1-2 cycles of induction chemotherapy or CR duration <3 months) by MCL-1 dependence. CRi = CR with incomplete recovery; PR = Partial Remission; MLFS = Morphologic Leukemia-Free State; NR = No Response; N/A = No response assessment due to early death.

**
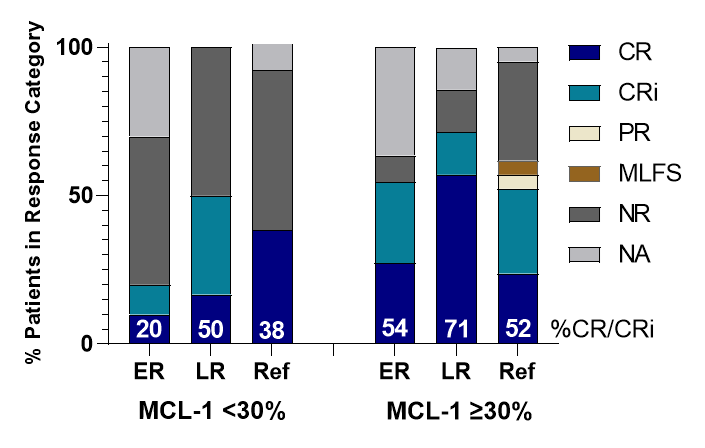
**

**Supplementary Figure 2. Clinical outcomes** **of alvocidib in combination with cytarabine and mitoxantrone in relapsed/refractory and newly diagnosed high-risk acute myeloid leukemia.**


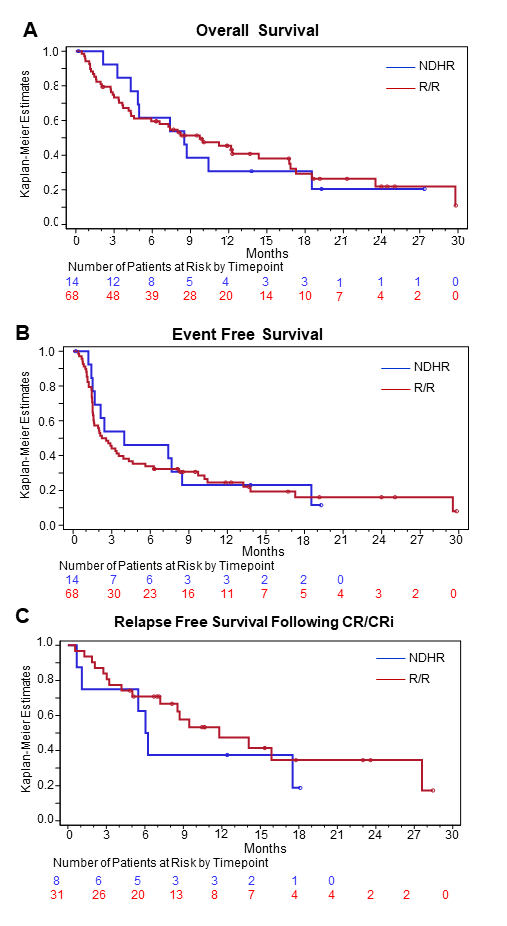
Overall survival (A), event free survival (B), and relapse free survival following CR/CRi (C) in R/R and NDHR AML patients from Day 1 of treatment up to death, relapse after CR, or no response to treatment.

**Supplementary Figure 3. Gene matrix of genomic biomarkers of response to of alvocidib in combination with cytarabine and mitoxantrone.** A heatmap showing the frequency of baseline mutations determined for CR versus those without a CR in patients treated with ACM. Color legends to the right represent the genomic class of all the patients as per Papaemmanuil et al.^6^

*Genomically defined secondary AML as defined in Lindsley et al.^7^

**Genomic Classification as defined in Papaemmanuil et al.^6^


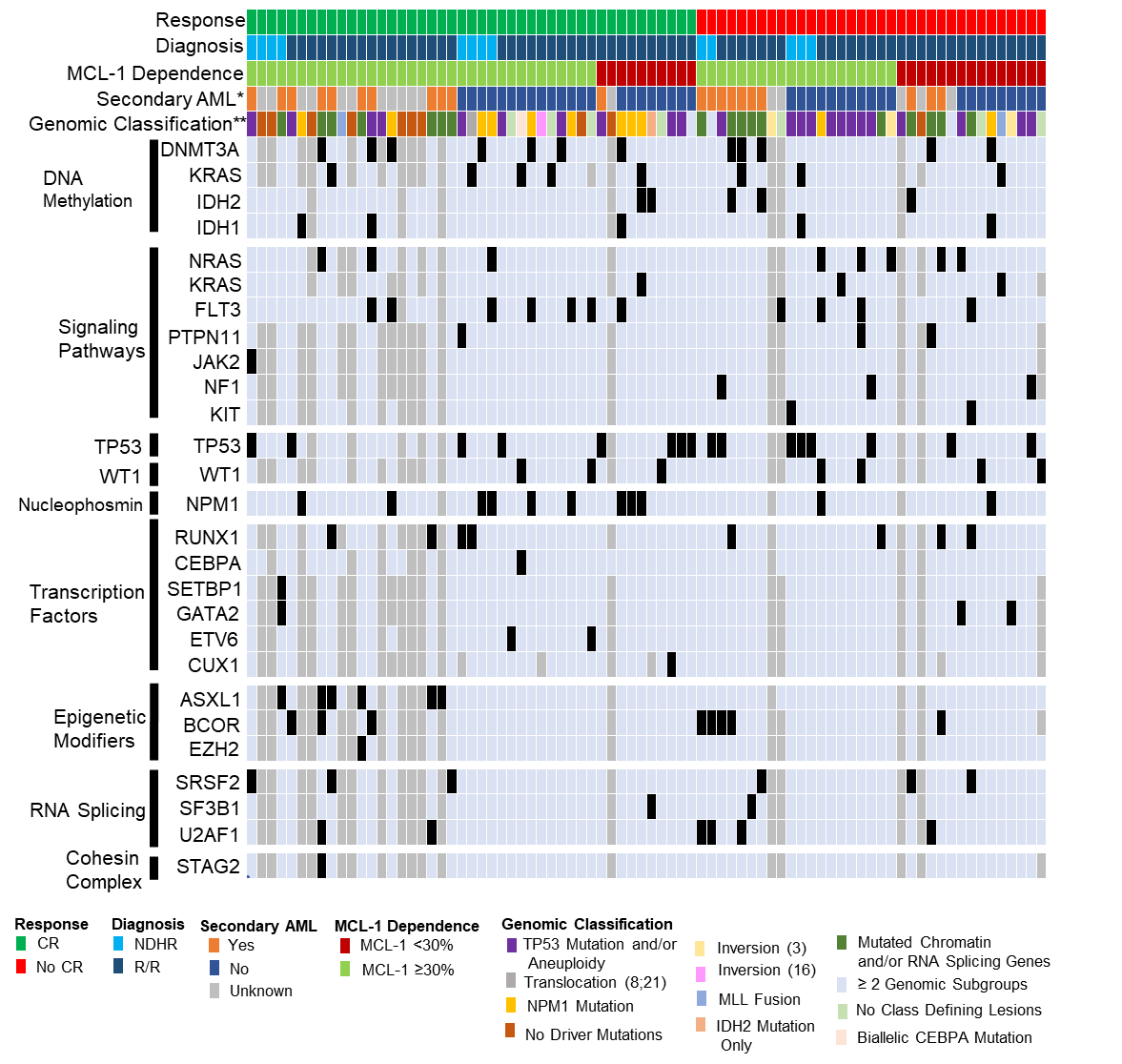

**Supplementary Figure 4.** **MCL-1 Dependence (>30% MCL-1 priming) Enriches for Clinical Responders in R/R AML**. Bootstrap resampling with replacement (1,000 replications) was performed on both the MCL-1 <30% cohort (n=25) and MCL-1 ≥30% cohort (n=43) with R/R AML. The count per sampling of clinical responders (CR, CRi, PR, and MLFS) vs non responders was compared by unpaired t-test to assess differences in the group means (A) <30% MCL-1 priming (CR = 11.6, NR = 17.39, *P*< .0001) and (B) ≥30% MCL-1 priming (CR = 29.05, NR = 13.95, *P*< .000001).

**Supplementary Table 1 Response Rate by MCL-1 Dependence**

|  | **Intention-To-Treat** | | | | | | | **Evaluable** | | | | | | |
| --- | --- | --- | --- | --- | --- | --- | --- | --- | --- | --- | --- | --- | --- | --- |
| **Response Characteristics** | **MCL-1**  **<15%**  **(n=15)** | **MCL-1**  **15-<30%**  **(n=14)** | **MCL-1**  **30-<40%**  **(n=14)** | **MCL-1**  **≥40%**  **(n=25)** | **MCL-1 <30%**  **(N=29)** | **MCL-1 ≥30%**  **(N=39)** | **NDHR**  **MCL-1**  **≥30%**  **(n=14)** | **MCL-1**  **<15%**  **(n=15)** | **MCL-1**  **15-<30%**  **(n=13)** | **MCL-1**  **30-<40%**  **(n=14)** | **MCL-1**  **≥40%**  **(n=23)** | **MCL-1 <30%**  **(N=28)** | **MCL-1 ≥30%**  **(N=37)** | **NDHR**  **MCL-1**  **≥30%**  **(n=13)** |
| CR, n (%) | 6 (40) | 1 (7) | 4 (29) | 8 (32) | 7 (24) | 12 (31) | 6 (43) | 6 (40) | 1 (8) | 4 (29) | 8 (35) | 7 (25) | 12 (32) | 6 (46) |
| CRi (%) | 1 (7) | 2 (14) | 5 (36) | 5 (20) | 3 (10) | 10 (26) | 2 (14) | 1 (7) | 2 (15) | 5 (36) | 5 (22) | 3 (11) | 10 (27) | 2 (15) |
| CRc (CR+CRi), n (%) | 7 (47) | 3 (21) | 9 (64) | 13 (52) | 10 (34) | 22 (56) | 8 (57) | 7 (47) | 3 (23) | 9 (64) | 13 (57) | 10 (36) | 22 (59) | 8 (62) |
| PR, n (%) | 0 | 0 | 0 | 1 (4) | 0 | 1 (3) | 1 (7) | 0 | 0 | 0 | 1 (4) | 0 | 1 (3) | 1 (8) |
| MLSF, n (%) | 0 | 0 | 1 (7) | 0 | 0 | 1 (3) | 0 | 0 | 0 | 1 (7) | 0 | 0 | 1 (3) | 0 |
| NA | 0 (0) | 4 (29) | 0 | 6 (24) | 4 (14) | 6 (15) | 1 (7) | 0 (0) | 3 (23) | 0 (0) | 4 (17) | 3 (11) | 4 (11) | 0 |
| NR | 8 (53) | 7 (50) | 4 (29) | 5 (20) | 15 (52) | 9 (23) | 4 (29) | 8 (53) | 7 (54) | 4 (29) | 5 (22) | 15 (54) | 9 (24) | 4 (31) |
| Overall Response Rate (CR+CRi+PR+MLFS) | 7 (47) | 3 (21) | 10 (71) | 14 (56) | 10 (34) | 24 (62) | 9 (64) | 7 (47) | 3 (23) | 10 (71) | 14 (61) | 10 (36) | 24 (65) | 9 (69) |
| Non-evaluable, n (%) ^a^ | 0 (0) | 1 (7) | 0 (0) | 2 (8) | 1 (3) | 2 (5) | 1 (7) | - | - | - | - | - | - | - |
| **Overall** **CRc (CR+Cri), n (%)** | | | | | | | | | | | | | | |
| Age <60 years, CRc/n | 6/12 (50) | 2/12 (17) | 8/12 (67) | 10/21 (48) | 8/24 (33) | 18/33 (55) | 6/9 (67) | 6/12 (50) | 2/11 (18) | 8/12 (67) | 10/19 (53) | 8/23 (35) | 18/31 (58) | 6/8 (75) |
| Age >60 years, CRc/n | 1/3 (33) | 1/2 (50) | 1/2 (50) | 3/4 (75) | 2/5 (40) | 4/6 (67) | 2/5 (40) | 1/3 (33) | 1/2 (50) | 1/2 (50) | 3/4 (75) | 2/5 (40) | 4/6 (67) | 2/7 (40) |
| Refractory, CRc/n | 4/8 (50) | 1/5 (20) | 4/9 (44) | 7/12 (58) | 5/13 (38) | 11/21 (52) | N/A | 4/8 (50) | 1/5 (20) | 4/9 (44) | 7/12 (58) | 5/13 (38) | 11/21 (52) | N/A |
| Early Relapse, CRc/n | 1/4 (25) | 1/6 (17) | 3/3 (100) | 3/8 (38) | 2/10 (20) | 6/11 (55) | N/A | 1/4 (25) | 1/5 (20) | 3/3 (100) | 3/6 (50) | 2/9 (22) | 6/9 (67) | N/A |
| Late Relapse, CRc/n | 2/3 (67) | 1/3 (33) | 2/2 (100) | 3/5 (60) | 3/6 (50) | 5/7 (71) | N/A | 2/3 (67) | 1/3 (33) | 2/2 (100) | 3/5 (60) | 3/6 (50) | 5/7 (71) | N/A |
| Secondary AML, CRc/n | 0/1 (0) | 0/2 (0) | 0/1 (0) | 0/3 (0) | 0/3 (0) | 0/4 (0) | 6/10 (60) | 0/1 (0) | 0/2 (0) | 0/1 (0) | 0/3 (0) | 0/4 (0) | 0/4 (0) | 6/10 (60) |
| AML with MRC, CRc/n | 3/6 (50) | 1/5 (20) | 2/5 (40) | 2/6 (33) | 4/11 (36) | 4/11 (36) | 5/10 (50) | 3/6 (50) | 1/5 (20) | 2/5 (40) | 2/6 (33) | 4/11 (36) | 4/11 (36) | 5/10 (50) |
| Genomically-defined Secondary AML, CRc/n | 1/3 (33) | 0/1 (0) | 3/4 (75) | 3/5 (60) | 1/4 (25) | 6/9 (67) | 2/4 (50) | 1/3 (33) | 0/1 (0) | 3/4 (75) | 3/5 (60) | 1/4 (25) | 6/9 (67) | 2/4 (50) |

CR= complete response; CRi= complete remission with incomplete recovery; MCL-1= myeloid cell luekemia-1; MLSF= morphologic leukemia-free state; NDHR= newly diagnosed high-risk; N/A= not available; NR= non-responsive

**Supplementary Table 2 Response Rate by MCL-1 Dependence Subdivided by Disease State**

|  | **Intention-to-Treat** | | | | | | | **Evaluable** | | | | | | |
| --- | --- | --- | --- | --- | --- | --- | --- | --- | --- | --- | --- | --- | --- | --- |
| **Response Characteristics** | **MCL-1**  **<15%**  **(n=15)** | **MCL-1**  **15-<30%**  **(n=14)** | **MCL-1**  **30-<40%**  **(n=14)** | **MCL-1**  **≥40%**  **(n=25)** | **MCL-1 <30%**  **(N=29)** | **MCL-1 ≥30%**  **(N=39)** | **NDHR**  **MCL-1**  **≥30%**  **(n=14)** | **MCL-1**  **<15%**  **(n=15)** | **MCL-1**  **15-<30%**  **(n=13)** | **MCL-1**  **30-<40%**  **(n=14)** | **MCL-1**  **≥40%**  **(n=23)** | **MCL-1 <30%**  **(N=28)** | **MCL-1 ≥30%**  **(N=37)** | **NDHR**  **MCL-1**  **≥30%**  **(n=13)** |
| **Refractory,** **n (%)** | | | | | | | | | | | | | | |
| CR/n | 4/8 (50) | 1/5 (20) | 1/9 (11) | 4/12 (33) | 5/13 (38) | 5/21 (24) | N/A | 4/8 (50) | 1/5 (20) | 1/9 (11) | 4/12 (33) | 5/13 (38) | 5/21 (24) | N/A |
| CRi/n | 0/8 (0) | 0/5 (0) | 3/9 (33) | 3/12 (25) | 0/13 (0) | 6/21 (29) | N/A | 0/8 (0) | 0/5 (0) | 3/9 (33) | 3/12 (25) | 0/13 (0) | 6/21 (29) | N/A |
| PR | 0/8 (0) | 0/5 (0) | 0/9 (0) | 1/12 (8) | 0/13 (0) | 1/21 (5) | N/A | 0/8 (0) | 0/5 (0) | 0/9 (0) | 1/12 (8) | 0/13 (0) | 1/21 (5) | N/A |
| **Early Relapse, n (%)** | | | | | | | | | | | | | | |
| CR/n | 1/4 (25) | 0/6 (0) | 1/3 (33) | 2/8 (25) | 1/10 (10) | 3/11 (27) | N/A | 1/4 (25) | 0/5 (0) | 1/3 (33) | 2/6 (33) | 1/9 (11) | 3/9 (33) | N/A |
| CRi/n | 0/4 (0) | 1/6 (17) | 2/3 (67) | 1/8 (13) | 1/10 (10) | 3/11 (27) | N/A | 0/4 (0) | 1/5 (20) | 2/3 (67) | 1/6 (17) | 1/9 (11) | 3/9 (33) | N/A |
| PR | 0/4 (0) | 0/6 (0) | 0/3 (0) | 0/8 (0) | 0/10 (0) | 0/11 (0) | N/A | 0/4 (0) | 0/5 (0) | 0/3 (0) | 0/6 (0) | 0/9 (0) | 0/9 (0) | N/A |
| **Late Relapse, n (%)** | | | | | | | | | | | | | | |
| CR/n | 1/3 (33) | 0/3 (0) | 2/2 (100) | 2/5 (40) | 1/6 (17) | 4/7 (57) | N/A | 1/3 (33) | 0/3 (0) | 2/2 (57) | 2/5 (40) | 1/6 (17) | 4/7 (57) | N/A |
| CRi/n | 1/3 (33) | 1/3 (33) | 0/2 (0) | 1/5 (20) | 2/6 (33) | 1/7 (14) | N/A | 1/3 (33) | 1/3 (33) | 0/2 (0) | 1/5 (20) | 2/6 (33) | 1/7 (14) | N/A |
| PR | 0/3 (0) | 0/3 (0) | 0/2 (0) | 0/5 (0) | 0/6 (0) | 0/7 (0) | N/A | 0/3 (0) | 0/3 (0) | 0/2 (0) | 0/5 (0) | 0/6 (0) | 0/7 (0) | N/A |
| **ELN-risk, n (%)** | | | | | | | | | | | | | | |
| Favorable, CRc/n | 2/2 (100) | 1/2 (50) | 0/0 (0) | 0/1 (0) | 3/4 (75) | 0/1 (0) | 3/3 (100) | 2/2 (100) | 1/2 (50) | 0/0 (0) | 0/1 (0) | 3/4 (75) | 0/1 (0) | 3/3 (100) |
| Intermediate, CRc/n | 2/4 (50) | 1/3 (33) | 7/8 (88) | 6/9 (67) | 3/7 (43) | 13/17 (76) | 2/2 (100) | 2/4 (50) | 1/3 (33) | 7/8 (88) | 6/9 (67) | 3/7 (43) | 6/17 (35) | 2/2 (100) |
| Adverse, CRc/n | 3/9 (33) | 1/6 (17) | 2/6 (33) | 7/11 (64) | 4/15 (27) | 9/17 (53) | 3/9 (33) | 3/9 (33) | 1/6 (17) | 2/6 (33) | 7/11 (64) | 4/15 (27) | 3/9 (33) | 3/8 (38) |
| Not Evaluable | 0/0 (0) | 0/3 (0) | 0/0 (0) | 0/4 (0) | 0/3 (0) | 0/4 (0) | 0/0 (0) | 0/0 (0) | 0/2 (0) | 0/0 (0) | 0/2 (0) | 0/2 (0) | 0/2 (0) | 0/0 (0) |
| **SWOG Cytogenetics Risk, n (%)** | | | | | | | | | | | | | | |
| Favorable, CRc/n | 0/0 (0) | 0/0 (0) | 0/0 (0) | 0/1 (0) | 0/0 (0) | 0/1 (0) | 1/1 (0) | 0/0 (0) | 0/0 (0) | 0/0 (0) | 0/1 (0) | 0/0 (0) | 0/1 (0) | 1/1 (0) |
| Intermediate, CRc/n | 3/7 (43) | 2/3 (67) | 4/4 (100) | 8/11 (73) | 5/10 (50) | 12/15 (80) | 4/4 (100) | 3/7 (43) | 2/3 (67) | 4/4 (100) | 8/11 (73) | 5/10 (50) | 12/15 (80) | 4/4 (100) |
| Unfavorable, CRc/n | 4/7 (57) | 1/6 (17) | 1/6 (17) | 3/6 (50) | 5/13 (38) | 4/12 (33) | 3/9 (33) | 4/7 (57) | 1/6 (17) | 1/6 (17) | 3/6 (50) | 5/13 (38) | 4/12 (33) | 3/8 (25) |
| Unknown, CRc/n | 0/1 (0) | 0/2 (0) | 4/4 (100) | 2/3 (67) | 0/3 (0) | 6/7 (86) | 0/0 (0) | 0/1 (0) | 0/2 (0) | 4/4 (100) | 2/3 (67) | 0/3 (0) | 6/7 (86) | 0/0 (0) |

CR= complete remission; CRi= complete remission with incomplete recovery; ELN= European LeukemiaNet; MCL-1= myeloid cell luekemia-1; MDS= myelodysplastic syndrome; MLSF= morphologic leukemia-free state; MRC= myelodysplasia-related changes; NDHR= newly diagnosed high-risk; PR= partial remission; SWOG=Southwestern Oncology Group; t-AML= treatment related AML

**Supplementary Table 3 Survival Analyses By MCL-1 Cohort**

| **Survival Measurement** | **MCL-1**  **<15%**  **months**  **N=15** | **MCL-1**  **15%-<30%**  **months**  **N=14** | **MCL-1**  **30-<40%**  **months**  **N=14** | **MCL-1**  **≥40%**  **months**  **N=25** | **NDHR**  **months**  **N=14** | **MCL-1**  **<30%**  **months**  **N=29** | **MCL-1**  **≥30%**  **months**  **N=39** |
| --- | --- | --- | --- | --- | --- | --- | --- |
| Median follow-up | 7.3 | 2.4 | 9.6 | 10.0 | 8.0 | 6.3 | 9.7 |
| Median OS  [95% CI] | NA  [4.1,NA] | 2.8  [1.4,12.2] | 16.9  [3.4,NA] | 10.0  [3.4,17.3] | 8.5  [4.3,18.5] | 7.4  [2.8,NA] | 11.2  [4.3,17.3] |
| Median EFS  [95% CI] | 2.2  [1.0,NA] | 1.5  [1.4,1.9] | 7.0  [1.6,NA] | 3.4  [1.4,6.3] | 4.0  [1.5,8.5] | 1.6  [1.5,2.2] | 3.5  [1.6,9.7] |
| Median RFS following CR/CRi  [95% CI] | NA  [NA,NA] | 7.2  [0.6,NA] | 11.8  [1.3,NA] | 8.7  [2.8,15.9] | 6.1 months  [0.7,NA] | NA  [0.6,NA] | 9.5  [3.2,27.6] |

CI= confidence interval; CR= complete remission; CRi= complete remission with incomplete recovery; EFS= event-free survival; MCL-1= myeloid cell luekemia-1; NA= not available; NDHR= newly diagnosed high-risk; OS= overall survival; RFS= relapse-free survival

No significant differences were detected between the MCL-1 <30% and MCL-1 ≥30% groups for OS, EFS, or RFS.

**Supplementary Table 4 Survival and Median Follow-up for Refractory, Early Relapse and Late Relapse in patients with Relapsed/Refractory AML in Stage 1**

|  | **All Stage 1**  **months** | | | **MCL-1 <30%**  **months** | | | **MCL-1 ≥30%**  **months** | | |
| --- | --- | --- | --- | --- | --- | --- | --- | --- | --- |
| Survival Measurement | Refractory  N=34 | Early Relapse  N=21 | Late Relapse  N=13 | Refractory  N=13 | Early Relapse  N=10 | Late Relapse  N=6 | Refractory  N=21 | Early Relapse  N=11 | Late Relapse  N=7 |
| Median follow-up | 9.1 | 3.4 | 8.3 | 6.3 | 2.1 | 8.0 | 11.9 | 5.9 | 14.4 |
| Median OS  [95% CI] | 12.2  [4.3,18.5] | 4.8  [1.4,8.0] | 14.4 [3.0,NA] | 8.2  [1.6,NA] | 3.6  [0.8,7.4] | NA  [2.8,NA] | 16.9  [4.3,23.5] | 5.9  [0.7,NA] | 14.4  [0.7,NA] |
| Median EFS  [95% CI] | 2.6  [1.5,8.2] | 1.9  [1.4,3.0] | 13.2 [1.5,NA] | 1.6  [1.2,NA] | 1.6  [0.8,2.0] | NA  [1.0,NA] | 3.9  [1.4,9.7] | 3.0  [0.7,10.2] | 13.2  [0.7,NA] |
| Median RFS following CR/CRi  [95% CI] | 9.5  [5.0,NA] | 2.1  [0.6,NA] | 14.1 [3.0,NA] | NA  [7.2,NA] | 0.6  [NA,NA] | NA  [NA,NA] | 9.5  [3.2,NA] | 5.4  [1.3,NA] | 14.1  [3.0,NA] |

CI= confidence interval; CR= complete remission; CRi= complete remission with incomplete recovery; EFS= event-free survival; MCL-1= myeloid cell luekemia-1; NA= not available; OS= overall survival; RFS= relapse-free survival

**Supplementary Table 5 Treatment-Emergent Grade ≥3 Non-Hematologic Toxicities**

| **Toxicity, n (%)** | **ACM**  **Relapsed/**  **Refractory**  **(N=68)** | **ACM**  **NDHR**  **(N=14)** | **ACM**  **Total**  **(N=82)** |
| --- | --- | --- | --- |
| **Cardiac disorders** |  |  |  |
| Left ventricular dysfunction | 0 | 2 (14) | 2 (2) |
| Arrhythmia | 1 (2) | 0 | 1 (1) |
| Cardiac failure | 1 (2) | 0 | 1 (1) |
| Mitral valve incompetence | 1 (2) | 0 | 1 (1) |
| Sinus tachycardia | 1 (2) | 0 | 1 (1) |
| Supraventricular tachycardia | 1 (2) | 0 | 1 (1) |
| Tachycardia | 1 (2) | 0 | 1 (1) |
| **Gastrointestinal disorders** |  |  |  |
| Diarrhea | 9 (13) | 4 (29) | 13 (16) |
| Colitis | 4 (6) | 2 (14) | 6 (7) |
| **General disorders and administration site conditions** |  |  |  |
| Fatigue | 2 (3) | 1 (7) | 3 (3) |
| Oedema peripheral | 3 (4) | 0 | 3 (3) |
| Disease progression | 2 (3) | 0 | 2 (2) |
| Mucosal inflammation | 1 (2) | 0 | 1 (1) |
| Chest pain | 1 (1) | 0 | 1 (1) |
| General physical health deterioration | 1 (1) | 0 | 1 (1) |
| Pain | 1 (1) | 0 | 1 (1) |
| **Hepatobiliary disorders** |  |  |  |
| Hyperbilirubinaemia | 2 (3) | 1 (7) | 3 (3) |
| Hypertransaminasaemia | 1 (1) | 0 | 1 (1) |
| Liver injury | 1 (1) | 0 | 1 (1) |
| **Immune system disorders** |  |  |  |
| Cytokine release syndrome | 2 (3) | 1 (7) | 3 (3) |
| **Injury, poisoning and procedural complications** |  |  |  |
| Allergic transfusion reaction | 1 (1) | 0 | 1 (1) |
| Transfusion reaction | 1 (1) | 0 | 1 (1) |
| **Infections and infestations** |  |  |  |
| Sepsis | 11 (16) | 3 (21) | 14 (17) |
| Pneumonia | 5 (7) | 2 (14) | 7 (9) |
| Lung infection | 5 (7) | 1 (7) | 6 (%) |
| Bacteraemia | 3 (4) | 0 | 3 (4) |
| Device related infection | 3 (4) | 0 | 3 (4) |
| Staphylococcal bacteraemia | 3 (4) | 0 | 3 (4) |
| Anorectal infection | 1 (2) | 2 (14) | 3 (4) |
| Enterococcal infection | 3 (4) | 0 | 3 (4) |
| Septic shock | 2 (3) | 0 | 2 (2) |
| Abscess neck | 1 (2) | 0 | 1 (1) |
| Alpha haemolytic streptococcal infection | 1 (2) | 0 | 1 (1) |
| BK virus infection | 1 (2) | 0 | 1 (1) |
| Bacterial sepsis | 1 (2) | 0 | 1 (1) |
| Clostridium difficile colitis | 1 (2) | 0 | 1 (1) |
| Enterobacter bacteraemia | 0 | 1 (7) | 1 (1) |
| Enterococcal bacteraemia | 1 (2) | 0 | 1 (1) |
| Enterococcal sepsis | 1 (2) | 0 | 1 (1) |
| Enterocolitis infectious | 2 (3) | 0 | 2 (2) |
| Enterocolitis viral | 1 (2) | 0 | 1 (1) |
| Escherichia infection | 1 (2) | 0 | 1 (1) |
| Fungal infection | 1 (2) | 0 | 1 (1) |
| Herpes simplex | 1 (2) | 0 | 1 (1) |
| Klebsiella infection | 1 (2) | 0 | 1 (1) |
| Klebsiella sepsis | 1 (2) | 0 | 1 (1) |
| Neutropenic sepsis | 1 (2) | 0 | 1 (1) |
| Pharyngitis streptococcal | 1 (2) | 0 | 1 (1) |
| Pilonidal cyst | 1 (2) | 0 | 1 (1) |
| Pneumonia fungal | 1 (2) | 0 | 1 (1) |
| Pneumonia legionella | 0 | 1 (7) | 1 (1) |
| Pneumonia parainfluenzae viral | 1 (2) | 0 | 1 (1) |
| Pneumonia viral | 1 (2) | 0 | 1 (1) |
| Respiratory syncytial virus infection | 1 (2) | 0 | 1 (1) |
| Sinusitis | 1 (2) | 0 | 1 (1) |
| Skin infection | 1 (2) | 0 | 1 (1) |
| Staphylococcal infection | 1 (2) | 0 | 1 (1) |
| Stomatococcal infection | 1 (2) | 0 | 1 (1) |
| Streptococcal bacteraemia | 1 (2) | 0 | 1 (1) |
| Tooth infection | 1 (2) | 0 | 1 (1) |
| **Investigations** |  |  |  |
| White blood cell count decreased | 25 (37) | 4 (29) | 29 (35) |
| Platelet count decreased | 15 (22) | 4 (29) | 19 (23) |
| Lymphocyte count decreased | 12 (18) | 4 (29) | 16 (20) |
| Neutrophil count decreased | 12 (18) | 1 (7%) | 13 (16) |
| Aspartate aminotransferase increased | 10 (15) | 1 (7) | 11 (13) |
| Alanine aminotransferase increased | 8 (12) | 1 (7) | 9 (11) |
| Blood bilirubin increased | 2 (3) | 1 (7) | 3 (4) |
| Blood lactate dehydrogenase increased | 2 (3) | 0 | 2 (2) |
| Electrocardiogram QT prolonged | 2 (3) | 0 | 2 (2) |
| Alanine aminotransferase abnormal | 1 (2) | 0 | 1 (1) |
| Aspartate aminotransferase abnormal | 1 (2) | 0 | 1 (1) |
| Blood creatinine increased | 1 (2) | 0 | 1 (1) |
| Blood fibrinogen decreased | 1 (2) | 0 | 1 (1) |
| Blood phosphorus decreased | 0 | 1 (7) | 1 (1) |
| Blood urea increased | 1 (2) | 0 | 1 (1) |
| Bronchoscopy | 1 (2) | 0 | 1 (1) |
| Ejection fraction decreased | 1 (2) | 0 | 1 (1) |
| Oxygen saturation decreased | 1 (2) | 0 | 1 (1) |
| Platelet count abnormal | 1 (2) | 0 | 1 (1) |
| Prothrombin time prolonged | 1 (2) | 0 | 1 (1) |
| **Metabolism and nutrition disorders** |  |  |  |
| Hypokalaemia | 18 (26) | 2 (14) | 20 (24) |
| Tumour lysis syndrome | 16 (24) | 3 (21) | 19 (23) |
| Hypophosphataemia | 14 (21) | 3 (21) | 17 (21) |
| Hypocalcaemia | 5 (7) | 3 (21) | 8 (10) |
| Decreased appetite | 5 (7) | 0 | 5 (5) |
| Hyperglycaemia | 3 (4) | 2 (14) | 5 (5) |
| Hypoalbuminaemia | 4 (6) | 1 (7) | 5 (5) |
| Hyponatraemia | 4 (6) | 0 | 4 (4) |
| Hyperkalaemia | 1 (2) | 0 | 1 (1) |
| Hypomagnesaemia | 0 | 1 (7) | 1 (1) |
| **Musculoskeletal and connective tissue disorders** |  |  |  |
| Back pain | 2 (3) | 0 | 2 (2) |
| Muscular weakness | 0 | 1 (7) | 1 (1) |
| **Nervous system disorders** | 5 (6) | 0 | 5 (6) |
| Syncope | 2 (3) | 0 | 2 (2) |
| Depressed level of consciousness | 1 (2) | 0 | 1 (1) |
| Dizziness | 1 (2) | 0 | 1 (1) |
| Neuropathy peripheral | 1 (2) | 0 | 1 (1) |
| **Psychiatric disorders** |  |  |  |
| Depression | 1 (2) | 0 | 1 (1) |
| Insomnia | 1 (2) | 0 | 1 (1) |
| Suicide attempt | 1 (2) | 0 | 1 (1) |
| **Renal and urinary disorders** |  |  |  |
| Acute kidney injury | 2 (3) | 1 (7) | 3 (4) |
| **Respiratory, thoracic and mediastinal disorders** |  |  |  |
| Hypoxia | 4 (6) | 1 (7) | 5 (6) |
| Pulmonary oedema | 2 (3) | 1 (7) | 3 (4) |
| Dyspnoea | 0 | 2 (14) | 2 (2) |
| Acute respiratory distress syndrome | 1 (2) | 0 | 1 (1) |
| Acute respiratory failure | 1 (2) | 0 | 1 (1) |
| Laryngeal inflammation | 1 (2) | 0 | 1 (1) |
| Organizing pneumonia | 1 (2) | 0 | 1 (1) |
| Respiratory distress | 1 (2) | 0 | 1 (1) |
| Tachypnoea | 0 | 1 (7) | 1 (1) |
| **Skin and subcutaneous tissue disorders** |  |  |  |
| Rash maculo-papular | 1 (2) | 1 (7) | 2 (2) |
| Rash | 1 (2) | 0 | 1 (1) |
| **Surgical and medical procedures** |  |  |  |
| Central venous catheterisation | 1 (2) | 0 | 1 (1) |
| **Vascular disorders** |  |  |  |
| Hypotension | 5 (7) | 3 (21) | 8 (10) |
| Hypertension | 1 (2) | 0 | 1 (1) |
| Shock | 1 (2) | 0 | 1 (1) |

ACM= Alvocidib/Cytarabine/Mitoxantrone; CM=Cytarabine/Mitoxantrone; MedDRA= Medical Dictionary for Regulatory Activities (v19.1)

Entries are sorted alphabetically by System Organ Class and within System Organ Class by Preferred Term frequency in the ACM Total column; NDHR= newly diagnosed high-risk

**References:**

1. Dohner H, Estey E, Grimwade D, et al. Diagnosis and management of AML in adults: 2017 ELN recommendations from an international expert panel. Blood. 2017;129(4):424-447.

2. Zeidner JF, Lee DJ, Frattini M, et al. Phase I Study of Alvocidib Followed by 7+3 (Cytarabine + Daunorubicin) in Newly Diagnosed Acute Myeloid Leukemia. Clin Cancer Res. 2021;27(1):60-69.

3. Foight GW, Ryan JA, Gulla SV, Letai A, Keating AE. Designed BH3 peptides with high affinity and specificity for targeting Mcl-1 in cells. ACS Chem Biol. 2014;9(9):1962-1968.

4. Davidson A, Hinkely, DV. Bootstrap Methods and Their Application. Cambridge, United Kingdom: Cambridge University Press, 1997.

5. Dohner H, Estey EH, Amadori S, et al. Diagnosis and management of acute myeloid leukemia in adults: recommendations from an international expert panel, on behalf of the European LeukemiaNet. Blood. 2010;115(3):453-474.

6. Papaemmanuil E, Gerstung M, Bullinger L, et al. Genomic classification and prognosis in acute myeloid leukemia. N Engl J Med. 2016;374(23):2209-2221.

7. Lindsley RC, Mar BG, Mazzola E, et al. Acute myeloid leukemia ontogeny is defined by distinct somatic mutations. Blood. 2015;125(9):1367-1376.
